# Harnessing red grape pomace extract for sustainable broad-spectrum photoprotection in a reconstructed human epidermis model

**DOI:** 10.3389/fphar.2025.1744377

**Published:** 2026-01-05

**Authors:** Antonella Smeriglio, Marta Mangano, Mariarosaria Ingegneri, Annarita La Neve, Chiara Brenna, Luca Mastracci, Francesco Merlino, Giancarlo Tonon, Diego Bosco, Domenico Trombetta

**Affiliations:** 1 Department of Chemical, Biological, Pharmaceutical and Environmental Sciences, University of Messina, Viale Ferdinando Stagno d'Alcontres, Messina, Italy; 2 Prigen srl, Milan, Italy; 3 Pathology Unit, Department of Surgical Sciences and Integrated Diagnostics (DISC), University of Genova, Genova, Italy; 4 IRCCS Ospedale Policlinico San Martino, Genoa, Italy; 5 Univerlab srl, Milan, Italy; 6 Italbiotec s.r.l., Milan, Italy

**Keywords:** anti-inflammatory activity, antioxidant activity, circular economy, dermocosmetic formulation, grape pomace extract, photoprotection, polyphenols, reconstructed human epidermis

## Abstract

Red grape pomace, a major by-product of the winemaking industry, represents a sustainable source of bioactive polyphenols with high nutraceutical and cosmetic potential. In this study, a standardized grape pomace extract (GPE) was developed from quality-controlled dried pomace, compliant with European Pharmacopoeia requirements for heavy metals, microbiological purity, and residual moisture. Phytochemical profiling by LC-DAD-ESI-MS/MS revealed a complex composition dominated by anthocyanins—primarily malvidin-3-*O*-glucoside—alongside lignans, stilbenes, and flavonols. The extract showed a high total phenolic content (2.64 g GAE/100 g) and exhibited strong, concentration-dependent antioxidant activity, with the highest efficacy in FRAP and ORAC assays, and significant anti-inflammatory effects in protein denaturation and protease inhibition tests. GPE demonstrated excellent photochemical stability and was incorporated into a lipid-based cosmetic formulation (SS + GPE) to assess its safety and photoprotective efficacy in a reconstructed human epidermis (RHE) model exposed to UVA (26 J/cm^2^) and UVB (1.5 J/cm^2^) radiation. Both GPE and SS + GPE treatments markedly reduced ROS, NO, and IL-1α production (up to 60%–65% reduction vs. irradiated control) while maintaining cell viability above 90%. Histological analysis further confirmed the structural protection of the epidermis, showing fewer dyskeratotic cells, restored intercellular cohesion, and normalization of keratinization. Overall, these results demonstrate that GPE is a safe, photostable, and multifunctional bioactive extract capable of counteracting UV-induced oxidative and inflammatory damage. This study provides a comprehensive and sustainable strategy for the upcycling of red grape pomace into high value dermocosmetic formulations with proven broad-spectrum photoprotective efficacy.

## Introduction

Grapes represent one of the most widespread and appreciated fruits worldwide (García-Lomillo and González-SanJosé, 2017). In addition to direct consumption, they are widely used in the production of numerous derivatives, including wine, jams, and juices. Viticulture constitutes one of the most relevant agro-economic activities globally, with an annual production of grapes exceeding 60 million tons, more than 20 million of which originate from Europe (FAO, 2024; Scoma et al., 2016).

Approximately 75% of global grape production is destined for winemaking, with a total estimated output of about 27 billion liters of wine per year (Zhu et al., 2015; Amienyo et al., 2014). The species most employed for this purpose is *Vitis vinifera* L. (Devesa-Rey et al., 2011). In recent years, alongside the increase in wine consumption, a significant rise in the amount of by-products generated by the wine industry has been recorded, particularly grape pomace (Beres et al., 2017).

Grape pomace derives from the pressing and fermentation of the must and mainly consists of skins and seeds, whose composition varies according to the vinification method. In red wine production, must and pomace ferment together, allowing the extraction of pigments such as anthocyanins, which are responsible for the characteristic coloration (Amienyo et al., 2014). In contrast, in white wine production, fermentation involves only the juice, leaving pomace as a solid residue (Dwyer et al., 2014).

Globally, millions of tons of grape pomace are produced annually (OIV, 2024), representing a major management challenge, especially for large-scale producers, due to the stringent environmental regulations governing the disposal of such residues (Muhlack et al., 2018). Traditional practices such as incineration or direct disposal are problematic because of the high content of phenolic compounds, which lower the pH, slow down biodegradation, and may cause water pollution, unpleasant odors, and the attraction of insects (Dwyer et al., 2014; Christ and Burritt, 2013).

In parallel with these environmental concerns, several studies have highlighted that grape pomace is a valuable source of bioactive compounds. It has been estimated that only 30%–40% of the phytochemicals present in grapes are transferred to wine during vinification, while the remaining fraction remains concentrated in the solid residues. During this process, most soluble nutrients such as sugars, organic acids, and water-soluble vitamins are extracted into the must, whereas the pomace retains the majority of phenolic compounds together with structural polysaccharides, lipids, and minerals (Peixoto et al., 2018; Milinčić et al., 2021).

The growing attention toward sustainability and the reduction of environmental impact has therefore stimulated the search for circular valorization strategies. Numerous studies have demonstrated that grape pomace can be reused in different application areas—from feed and biofuel production to the recovery of polyphenols intended for the food, nutraceutical, and cosmetic industries (Fontana et al., 2017; Onache et al., 2022).

From a compositional point of view, grape pomace is particularly rich in phenolic acids, flavonoids, and anthocyanins, as well as in lipids and structural polymers such as cellulose and hemicellulose (Eyiz et al., 2020; Romero-Díez et al., 2019; Ianni and Martino, 2020; Fan et al., 2020; Jin et al., 2019). These compounds, known for their antioxidant properties, exert numerous biological effects, including anti-inflammatory, anticancer, cardioprotective, antithrombotic, and even hair growth–stimulating activities (Hübner et al., 2019). The antioxidant activity, largely attributable to phenolic compounds, also contributes to a photoprotective effect, opening interesting prospects for their use in cosmetic formulations, particularly sunscreens (Renaud and de Lorgeril, 1992).

Considering this evidence, grape pomace can be considered a strategic raw material: on one hand, a management challenge for the wine industry; on the other, a versatile resource for the production of bioactive ingredients with high economic and functional value.

Within this framework, the present study aimed to perform an integrated phytochemical and biological characterization of a hydroalcoholic extract obtained from red *V*. *vinifera* grape pomace, in order to valorize this winemaking by-product as a potential nutraceutical and dermopharmacological ingredient.

To the best of our knowledge, pharmacopeia-compliant and fully standardized grape pomace extracts have not yet been investigated through an integrated chemical and biological approach encompassing antioxidant, anti-inflammatory, and photoprotective evaluations, including assessment in a reconstructed human epidermis (RHE) model. Overall, this study provides new insights into the multifunctional potential of grape pomace extracts as sustainable modulators of oxidative stress, with promising applications in preventive nutraceuticals and functional cosmetics.

## Materials and methods

### Raw material

Fresh grape pomace from red *V. vinifera* grapes was collected immediately after pressing and alcoholic fermentation at a local winery (Casa Vinicola Criserà SRL, Reggio Calabria, Italy). The material had not been previously reused or subjected to any additional treatments. After collection, the pomace was placed in food-grade containers, transported under refrigerated conditions, and processed within 24 h to prevent compositional degradation.

### Quality control

#### Loss on drying

The residual moisture content of the grape pomace was determined according to the European Pharmacopoeia Ph. Eur. (2023a). Approximately 1.0 g of the powdered sample was accurately weighed into pre-dried inert weighing capsules and dried in a thermostatically controlled oven until constant weight. The constant mass condition was defined as two consecutive weighings, performed at 30-min intervals, differing by no more than 0.5 mg. After drying, samples were cooled to room temperature in a desiccator and reweighed. The percentage loss on drying, representing the water content, was calculated as the difference between the initial and final masses of the sample relative to its initial mass, and expressed as % (m/m):

$$\text{Loss on drying }\left(\%\;m/m\right)=\frac{m_{\text{before}}-m_{\text{after}}}{m_{\text{before}}}\times 100$$

#### Heavy metals

The content of toxic elemental impurities (As, Cd, Hg, and Pb) was determined according to the European Pharmacopoeia Ph. Eur. (2023b) using Inductively Coupled Plasma–Optical Emission Spectrometry (ICP-OES). Prior to analysis, all glassware was acid-washed with nitric acid (10 g/L) and rinsed with reagent-grade water to prevent contamination.

Approximately 0.5 g of finely powdered grape pomace was weighed into Teflon digestion vessels and treated with 4 mL of hydrochloric acid and 6 mL of nitric acid, both certified “heavy-metal free.” Samples were digested using a microwave-assisted program (80% power for 15 min, 100% for 5 min, and 80% for 20 min) until clear and colorless solutions were obtained. After cooling, digests were transferred to 50-mL volumetric flasks and brought to volume with reagent-grade water. A reagent blank, prepared under identical conditions but without sample, was included in each run.

The elemental analysis was performed using an external calibration curve prepared with certified multi-element standards at the pharmacopeial wavelengths (As: 193.7 nm; Cd: 214.4 nm; Hg: 324.7 nm; Pb: 220.3 nm). System suitability was verified by ensuring (i) transparency of the digest solution and (ii) instrumental response of a mid-range standard within ±20% of the nominal value. The concentration of each metal was expressed as mg/kg (ppm) on a dry-weight basis after blank correction.

#### Microbiological quality

Microbiological enumeration was performed in accordance with the European Pharmacopoeia Ph. Eur. (2023c). Briefly, 10 g of powdered grape pomace were suspended in 90 mL of sterile buffered peptone water (pH 6–8) containing polysorbate 80 (1 g/L) to facilitate the dispersion of phenolic components and to reduce potential antimicrobial interference. The resulting 1:10 (w/v) suspension was homogenized and subjected to membrane filtration through sterile filters with a pore size ≤0.45 µm. Each filter was then transferred aseptically onto the appropriate culture medium: Tryptic Soy Agar (TSA) for the determination of total aerobic microbial count (TAMC) and Sabouraud Dextrose Agar (SDA) for total yeasts and moulds count (TYMC). TSA plates were incubated at 30 °C–35 °C for 3–5 days, while SDA plates were incubated at 20 °C–25 °C for 5–7 days. After incubation, visible colonies were counted and results expressed as colony-forming units per gram of dry sample (CFU/g).

The presence of *Escherichia coli* and *Salmonella* spp. was assessed according to the procedures described in the European Pharmacopoeia (Ph. Eur. 2.6.13). For *E. coli*, 1 g of powdered pomace was suspended in 9 mL of Casein Soybean Digest Broth (TSB) and incubated at 30 °C–35 °C for 18–24 h (pre-enrichment phase). Subsequently, 1 mL of the culture was transferred into 100 mL of MacConkey broth and incubated at 42 °C–44 °C for 24–48 h for selective enrichment. After incubation, aliquots were streaked onto MacConkey agar plates and further incubated at 30 °C–35 °C for 18–72 h. The appearance of lactose-fermenting (pink to red) colonies was considered presumptive for *E. coli* and subjected to confirmatory biochemical identification.

For *Salmonella* spp., 10 g of sample were suspended in 90 mL of TSB and incubated at 30 °C–35 °C for 18–24 h (pre-enrichment), followed by selective enrichment in Rappaport–Vassiliadis (RV) broth (0.1 mL inoculum in 10 mL; 30 °C–35 °C, 18–24 h). Cultures were then streaked on Xylose Lysine Deoxycholate (XLD) agar and incubated at 30 °C–35 °C for 18–48 h. The presence of well-developed red colonies with or without a black center (H_2_S production) was considered indicative of *Salmonella* spp. and further confirmed through specific identification tests.

All culture media were subjected to growth-promotion and suitability testing prior to use. Samples were considered compliant when neither *E. coli* nor *Salmonella* spp. were detected.

### Extraction procedure and sample preparation

Phenolic compounds were extracted from dried grape pomace using a hydroalcoholic solvent system, following the method reported by Liu et al. (2024) with minor modifications. Briefly, ethanol (96%, v/v) and distilled water were mixed to obtain a final concentration of 60% ethanol (v/v), and the pH was adjusted to 2.0 using concentrated hydrochloric acid. Two grams of the dried and finely ground pomace were transferred into a 250 mL Erlenmeyer flask containing 100 mL of the hydroalcoholic solution. The mixture was stirred magnetically and heated to 60 °C to promote efficient solvent penetration and extraction of polyphenolic constituents. Once the target temperature was reached, heating was stopped, and the mixture was sonicated (200 W power 100% amplitude, 5 min) using a probe sonicator Model VC100 (Sonics and Materials Inc.) to further enhance the release of intracellular compounds. After cooling to room temperature, the suspension was centrifuged at 3500 rpm for 10 min at 4 °C to separate the solid residue. The supernatant was carefully collected and filtered (Whatman No. 2 filter paper, GE Healthcare) into a round-bottom flask. The filtrate was concentrated to dryness under reduced pressure using a rotary evaporator (Hei-VAP Core HL-G1, Heidolph, Schwabach, Germany) at a temperature not exceeding 40 °C to avoid degradation of thermolabile compounds.

The dried grape pomace extract (GPE) was weighed to calculate the extraction yield and stored in airtight amber glass containers, protected from light and humidity, until further phytochemical and biological analyses.

For analytical purposes, GPE was reconstituted in the same hydroalcoholic extraction mixture (ethanol/water, 60:40 v/v, pH 2). The resulting stock solution (100 mg/mL) was subsequently diluted in water to obtain the specific working concentrations required for the preliminary spectrophotometric phytochemical screening and for the *in vitro* cell-free antioxidant and anti-inflammatory assays (see specific sections for assay-dependent dilutions). For LC-DAD-ESI-MS/MS profiling, the extract was dissolved in the above hydroalcoholic extraction mixture at a final concentration of 1 mg/mL, filtered through a 0.22 µm nylon syringe filter, and then injected into the HPLC system.

### Phytochemical characterization

#### Determination of total phenolic content (TPC)

The total phenolic content was quantified using the Folin–Ciocalteu colorimetric assay, following the procedure described by Ingegneri et al. (2023) with minor modifications. Aliquots of 10 µL of GPE (0.625–10 mg/mL) were mixed with 100 µL of Folin–Ciocalteu reagent and 90 µL of distilled water in borosilicate tubes. After 3 min of incubation, 100 µL of 10% Na_2_CO_3_ were added to initiate the reaction. Samples were incubated for 60 min at room temperature (RT) in the dark, with gentle vortexing every 10 min. Absorbance was read at 785 nm using a UV–Vis microplate reader (Multiskan™ GO, Thermo Scientific, Waltham, MA, United States). A calibration curve was prepared using gallic acid as standard (75–600 μg/mL). Results were expressed as grams of gallic acid equivalents per 100 g of dry extract (g GAE/100 g DE).

#### Determination of total flavonoid content (TFC)

Total flavonoids were determined by the aluminum chloride colorimetric method, as described by Ingegneri et al. (2023). The assay is based on the formation of a stable complex between AlCl_3_ and the hydroxyl and carbonyl groups of flavonoids yielding an orange chromophore with maximum absorbance at 510 nm. Briefly, 50 µL of GPE (0.625–10 mg/mL) were mixed with 450 µL of deionized water and 30 µL of 5% NaNO_2_. After incubation for 5 min at RT, 60 µL of 10% AlCl_3_ were added. Following a second incubation of 6 min, 200 µL of 1 M NaOH and 210 µL of water were added to stop the reaction and develop the color. A corresponding blank was prepared by substituting the AlCl_3_ solution with an equal volume of deionized water, to correct for the intrinsic coloration of the extract and eliminate potential matrix interferences. Absorbance was measured at 510 nm using a UV–Vis spectrophotometer (Shimadzu UV-1601, Kyoto, Japan). Calibration was performed using rutin (125–1000 μg/mL), and results were expressed as grams of rutin equivalents (RE) per 100 g of DE.

#### Determination of total anthocyanins

Monomeric anthocyanins were quantified using the pH differential method (Lee et al., 2005). This technique exploits the structural transformation of anthocyanins at different pH values, resulting in a reversible color change measurable spectrophotometrically. Aliquots of 400 µL of the GPE were diluted in 2.8 mL of potassium chloride buffer (0.025 M, pH 1.0) and in 2.8 mL of sodium acetate buffer (0.4 M, pH 4.5), respectively. Absorbance was measured at 510 and 700 nm in both buffers using a UV–Vis spectrophotometer. The corrected absorbance (Abs) was calculated as follows:

$$\text{Abs}=\left[\left(A510-A700\text{ nm}\right)\text{pH }1\right]-\left[\left(A510-A700\text{ nm}\right)\text{pH }4.5\right]$$

Results were expressed as grams of Malvidin-3-*O*-glucoside equivalents (MGE) per 100 g of DE, using a molar absorptivity (ε) of 28,000 L·mol^-1^·cm^-1^ and a molecular weight (MW) of 493.43 g/mol.

#### Determination of flavan-3-ols

The content of flavan-3-ols was determined using the vanillin assay, as reported by Smeriglio et al. (2025a), after preliminary solid-phase extraction (SPE) purification. Two milliliters of GPE, diluted in 0.5 M sulfuric acid, were loaded onto Sep-Pak C18 cartridges (Waters, Milan, Italy), previously conditioned with methanol and water. The cartridges were washed with 5 mM sulfuric acid to remove interfering substances, then eluted with 5 mL of methanol. For the colorimetric reaction, 1 mL of the methanolic eluate was mixed with 6 mL of 4% vanillin in methanol and incubated for 10 min at 20 °C, followed by the addition of 3 mL of concentrated HCl and incubation for another 15 min in the dark. Absorbance was read at 500 nm. A calibration curve was constructed using catechin (125–500 μg/mL), and results were expressed as grams of catechin equivalents (CE) per 100 g of DE.

#### Determination of proanthocyanidins

Proanthocyanidins of GPE were quantified by acid hydrolysis followed by spectrophotometric detection, according to Smeriglio et al. (2025a). After SPE purification (as described above), the methanolic eluate (3 mL) was transferred into a 100 mL round-bottom flask protected from light. Ethanol (9.5 mL) and 12.5 mL of FeSO_4_·7H_2_O in concentrated HCl (300 mg/L) were added, and the mixture was refluxed for 50 min. After cooling, absorbance was measured at 550 nm, using a parallel sample not subjected to heating as blank. The concentration of proanthocyanidins was calculated using the molar extinction coefficient of cyanidin (ε = 34,700 L·mol^−1^·cm^−1^) and expressed as grams of cyanidin chloride equivalents per 100 g of dry extract (g CcE/100 g DE).

#### Polymerization index (PI)

The polymerization index was calculated according to Smeriglio et al. (2025a) as the ratio between monomeric flavan-3-ols and total proanthocyanidins. This index provides an indirect estimation of the mean degree of polymerization of condensed tannins: values > 1 indicate a predominance of monomeric forms (lower polymerization), whereas values < 1 reflect a higher contribution of oligomeric or polymeric structures.

### Polyphenolic profile by LC-DAD-ESI-MS/MS

The qualitative profile of polyphenolic compounds was determined using liquid chromatography coupled with diode array detection and electrospray ionization tandem mass spectrometry (LC-DAD-ESI-MS/MS) according to Smeriglio et al. (2025b). Analyses were performed on an Agilent 1200 HPLC system equipped with a photodiode array detector and an ion trap mass spectrometer (model 6320, Agilent Technologies, Santa Clara, CA, United States). Chromatographic separation was achieved on a Luna Omega PS C18 column (150 × 2.1 mm, 5 μm; Phenomenex, Torrance, CA, United States) maintained at 25 °C. The mobile phase consisted of (A) water containing 0.1% formic acid and (B) acetonitrile, delivered at a flow rate of 0.4 mL/min. The injection volume was 5 µL. The elution gradient was programmed as follows: 0% B (0–3 min), 3% B (3–9 min), 12% B (9–24 min), 20% B (24–33 min), 30% B (33–43 min), 50% B (43–66 min), 60% B (66–81 min), returning to 0% B (81–86 min) and held for 4 min for column re-equilibration. UV–Vis spectra were recorded between 190 and 600 nm, and chromatograms were acquired at 260, 292, 330, 370, and 520 nm to cover the major classes of phenolic compounds. Mass spectrometric analyses were carried out in both positive and negative ESI modes under the following conditions: capillary voltage 3.5 kV, nebulizer pressure 40 psi (nitrogen), drying gas temperature 350 °C, flow rate 9 L/min, and skimmer voltage 40 V. Full-scan spectra were acquired over the m/z range 90–1000, while MS/MS experiments were performed using collision-induced dissociation (CID) with a fragmentation amplitude of 1.2 V. Data acquisition and processing were performed using Agilent ChemStation (version B.01.03) and Trap Control (version 6.2) software. Compound identification was based on retention times, UV–Vis spectra, and MS/MS fragmentation patterns, compared with available reference standards of analytical grade and literature data. When standards were not available, tentative identification was achieved by matching spectral information with open-access databases such as SpectraBase^®^, PhytoHub, ReSpect, MassBank, and PubChem. The relative abundance of individual phenolics was expressed as peak area intensity in the total ion chromatogram (TIC).

### Antioxidant and anti-inflammatory activity

The antioxidant and anti-inflammatory activities of GPE were evaluated through a comprehensive panel of validated *in vitro* assays. Antioxidant potential was assessed using methods representative of both electron-transfer (ET) and hydrogen-atom transfer (HAT) mechanisms, including the 2,2-diphenyl-1-picrylhydrazyl radical scavenging assay (DPPH), the Trolox Equivalent Antioxidant Capacity (TEAC), the Ferric Reducing Antioxidant Power (FRAP), the Oxygen Radical Absorbance Capacity (ORAC), the β-carotene bleaching test (BCB), and the iron-chelating assay (ICA), as previously described by Ingegneri et al. (2023) and Smeriglio et al. (2025a). Anti-inflammatory activity was evaluated through two complementary *in vitro* models reproducing key mechanisms of inflammatory processes: albumin denaturation assay (ADA) and protease inhibition assay (PIA) in line with established procedures (Ingegneri et al., 2023). For each test, results were expressed as IC_50_ values (mean ± SD, n = 3), calculated from concentration–response curves using the Litchfield and Wilcoxon method with PHARM/PCS software (version 4; Consulting, Wynnewood, PA, United States).

#### DPPH

The DPPH assay was performed according to Ingegneri et al. (2023). A methanolic solution of DPPH (1 mM) was freshly prepared and protected from light. To 150 µL of this solution, 3.75 µL of the GPE (31.25–500 μg/mL) were added, and the mixture was incubated in the dark for 20 min at RT. Absorbance was measured at 517 nm using the same microplate reader reported in the section of *Determination of TPC*. Trolox (2.5–20 μg/mL) was used as a reference antioxidant.

#### FRAP

The FRAP assay was performed as reported by Ingegneri et al. (2023). The working FRAP reagent was prepared by mixing acetate buffer (300 mM, pH 3.6), 10 mM TPTZ in 40 mM HCl, and 20 mM FeCl_3_ in a 10:1:1 ratio. Ten microliters of GPE (15–240 μg/mL) were added to 200 µL of FRAP reagent, and the reaction mixture was incubated at 37 °C for 4 min in the dark. Absorbance was measured at 593 nm against a reagent blank using the same microplate reader reported in the section *Determination of TPC*. Trolox (1.25–10 μg/mL) served as reference standard.

#### TEAC

The TEAC assay was performed following Ingegneri et al. (2023). The ABTS^·+^ radical cation was generated by reacting 1.7 mM ABTS with 4.3 mM potassium persulfate and incubating for at least 12 h in the dark at room temperature. The resulting blue-green solution was diluted to an absorbance of 0.70 ± 0.02 at 734 nm. Ten microliters of GPE (15–240 μg/mL) were added to 200 µL of ABTS^·+^ solution and incubated for 6 min at room temperature. The decrease in absorbance at 734 nm was recorded using the same microplate reader reported in the section of *Determination of TPC*. Trolox (1.25–10 μg/mL) was used as a reference antioxidant.

#### ORAC

The ORAC assay was performed according to Ingegneri et al. (2023). Twenty microliters of GPE (0.78–12.5 μg/mL) were mixed with 120 µL of fluorescein solution (117 nM) and preincubated at 37 °C for 15 min. The reaction was initiated by adding 60 µL of AAPH (40 mM). Fluorescence decay was monitored every 30 s for 90 min (excitation 485 nm, emission 520 nm) using a FLUOstar Omega microplate reader (BMG LABTECH, Ortenberg, Germany). Trolox (0,25–2.0 μg/mL) served as reference standard.

#### ICA

The chelating ability of the extract toward Fe^2+^ ions was assessed using the ferrozine assay as described by Smeriglio et al. (2025a). Fifty microliters of FeCl_2_·4H_2_O (2 mM) were added to 100 µL of GPE (20–160 μg/mL), followed by 100 µL of ferrozine (5 mM) and 3 mL of deionized water. After incubation for 10 min at RT, absorbance was measured at 562 nm using the same microplate reader reported in the section of *Determination of TPC*. Ethylenediaminetetraacetic acid (EDTA, 1.5–12 μg/mL) was used as reference compound.

#### BCB

The antioxidant capacity in lipid systems was assessed using the β-carotene–linoleic acid bleaching method, according to Smeriglio et al. (2025a). An emulsion was prepared containing 250 µL of β-carotene solution (1 mg/mL in ethyl acetate), 4 µL of linoleic acid, and 40 µL of Tween 40. After solvent evaporation under nitrogen, the residue was emulsified in 5 mL of oxygenated deionized water. Five milliliters of the emulsion were mixed with 200 µL of the GPE (12.5–200 μg/mL) and incubated at 50 °C for 2 h under stirring. Absorbance was recorded every 20 min at 470 nm using the same microplate reader reported in the section of *Determination of TPC*. Butylated hydroxytoluene (BHT, 1 mg/mL) was used as standard.

#### ADA

The ability of the extract to prevent heat-induced protein denaturation was assessed using the bovine serum albumin (BSA), as described by Ingegneri et al. (2023). Briefly, reaction mixtures containing the GPE (125–2000 μg/mL), BSA solution (0.4% w/v), and phosphate-buffered saline (PBS, pH 5.3) were prepared in a volumetric ratio of 4:5:1. Diclofenac sodium (3–24 μg/mL) was used as reference anti-inflammatory drug. Samples were incubated at 70 °C for 30 min under gentle agitation, and the absorbance was measured at 595 nm using the microplate reader reported in the section *Determination of TPC*, both before and after heating. Thermal denaturation of albumin causes turbidity of the solution, resulting in an increase in absorbance, whereas anti-inflammatory agents reduce this effect by stabilizing the protein structure.

#### PIA

The inhibition of proteolytic activity was determined using trypsin as model enzyme, following the procedure reported by Ingegneri et al. (2023). Reaction mixtures were prepared by combining the GPE (62.5–1000 μg/mL), trypsin solution (12 μL, 10 μg/mL), and Tris–HCl buffer (20 mM, pH 7.5) to a final volume of 400 µL. Diclofenac sodium (5–80 μg/mL) served as positive control. After 10 min of preincubation at 37 °C, enzymatic hydrolysis was initiated by adding 200 µL of 0.8% (w/v) casein solution. The mixtures were then incubated for 20 min at 37 °C, and the reaction was stopped by adding 400 µL of 2 M perchloric acid to precipitate undigested proteins. Following centrifugation at 3500 *g* for 10 min, the absorbance of the supernatant, corresponding to the soluble peptides released by casein hydrolysis, was measured at 280 nm using the UV–Vis spectrophotometer reported in the section *Determination of TFC*. A decrease in absorbance relative to the enzyme control indicated inhibition of trypsin activity.

### Photostability, phototoxicity, and photoprotection

#### Photostability

The photostability of the GPE was evaluated following the procedure described by Tavares et al. (2020), with slight modifications. Two solutions of the extract (10 mg/mL) were prepared using either ethanol/water (60:40, v/v, pH 2.0) or propylene glycol, the latter being a common solvent in cosmetic formulations. For each solvent, 1 mL of the solution was placed in a 10 mL glass beaker (exposure surface: 4.5 cm^2^), and the solvent was evaporated under a gentle nitrogen stream to form a thin, homogeneous film. Control samples were kept in the dark under identical conditions. Irradiation was performed using a portable photostability unit (RTH469.1, Carl Roth GmbH + Co. KG., Karlsruhe, Germany) equipped with 15 W UVA (P373.1) and UVB (P851.1) lamps. The UV doses were adjusted to 26 J/cm^2^ (UVA) and 1.5 J/cm^2^ (UVB), as measured by a calibrated photometer (PCE-UV34, PCE Italia S.r.l., Lucca, Italy), to simulate an acute cumulative solar irradiation. After exposure, irradiated and control films were reconstituted (1:10 dilution) in their respective solvents, and the UV–Vis spectra were recorded in the 400–700 nm range—corresponding to the visible bands of the main chromophoric polyphenols (primarily anthocyanins), which represent the most reliable markers of pigment stability following UV exposure—using the UV–Vis spectrophotometer described in the section *Determination of TFC*.

Photostability was expressed as the percentage of residual absorbance area (AUC) of the irradiated sample compared with the corresponding unexposed control.

#### Cosmetic formulation

A high-protection sunscreen emulsion (SPF 50+) was developed as an oil-in-water (O/W) formulation to assess the photostability, safety, and photoprotective efficacy of GPE. The formulation was designed to ensure optimal skin compatibility and chemical stability of the incorporated bioactive plant complex. The emulsion contained an aqueous phase with glycerin and xanthan gum, and an oily phase with C12–15 alkyl benzoate, coco-caprylate, olive and grape seed oils, combined with organic UV filters (ethylhexyl triazone, diethylamino hydroxybenzoyl hexyl benzoate, bis-ethylhexyloxyphenol methoxyphenyl triazine, and disodium phenyl dibenzimidazole tetrasulfonate) providing broad-spectrum protection. Tocopherol acted as an antioxidant, while sodium stearoyl glutamate and Emulgin VL were used as emulsifiers.

The GPE was incorporated at a final concentration of 0.5% (w/w) as a 10% solution in propylene glycol, together with sodium hyaluronate (0.5%) and a preservative system (phenoxyethanol and ethylhexylglycerin). The pH was adjusted to 5.5 ± 0.1.

Both the GPE-enriched sunscreen formulation (SS + GPE) and the corresponding base formulation without GPE (SS), used as control, were developed and provided by Prigen S.r.l. (Milan, Italy). After 30 days of storage at 25 °C and 40 °C, no phase separation, colour change, or viscosity alteration was observed, confirming their physical and chemical stability and the compatibility of GPE within the cosmetic matrix.

#### Phototoxicity

Phototoxic potential was assessed using a RHE model (Episkin^®^, Lyon, France), according to the OECD (2019) and the method validated by the European Union Reference Laboratory for Alternatives to Animal Testing (European Commission/EURL-ECVAM, 2024).

RHE tissues (0.5 cm^2^) were equilibrated overnight at 37 °C and 5% CO_2_ in maintenance medium (SkinEthic™, SMM). The next day, 20 µL of GPE in propylene glycol (5 mg/mL), 20 mg of SS + GPE, and 20 µL of 3% ketoprofen in propylene glycol (positive control) were applied to the tissue surface under mesh supports.

After 18 h incubation, the meshes were removed, and one set of treated tissues was exposed to UVA irradiation (6 J/cm^2^ for 34 min) using the same UV apparatus described in the previous section, while the corresponding controls were kept unirradiated.

Cell viability was determined by the MTT assay. Tissues were rinsed with phosphate-buffered saline (PBS), incubated with MTT solution (0.5 mg/mL in SMM) for 3 h at 37 °C, and then extracted with a mixture of isopropanol and 40 mM HCl for 3 h under gentle shaking. The formazan content was quantified at 540 nm using the microplate reader described in the section *Determination of TPC*.

#### Photoprotection

The photoprotective efficacy of the GPE was investigated using the same RHE model under both UVA and UVB exposure conditions, following the protocol of Smeriglio et al. (2023). The extract (5 mg/mL in propylene glycol) was tested both alone and incorporated into an SPF 50+ sunscreen formulation (SS + GPE) at equivalent concentration. A placebo formulation (SS) and untreated tissues served as controls.

Tissues were divided into three groups: (i) UVA-irradiated (26 J/cm^2^), (ii) UVB-irradiated (1.5 J/cm^2^), and (iii) non-irradiated controls. Treatments (2 µL of GPE/propylene glycol or 2 mg of SS + GPE/SS) were applied to the tissue surface 1 h before irradiation.

After exposure, intracellular reactive oxygen species (ROS) were quantified by fluorescence using 2′,7′-dichlorodihydrofluorescein diacetate (DCFH_2_-DA, 50 µM). Tissues were incubated for 45 min in the dark, washed, and lysed in 1% Triton X-100 in PBS. Fluorescence (λ_ex_ 485 nm; λ_em_ 535 nm) was recorded using the fluorescence microplate reader described in the section *ORAC*.

Basolateral media were collected and stored at −20 °C for cytokine and nitric oxide analyses. Interleukin-1α (IL-1α) was quantified using a high-sensitivity human ELISA kit (RAB0269, Merck KGaA, Darmstadt, Germany), while nitrite levels were determined via the Griess reaction, using sodium nitrite as standard (0–15 µM), according to Smeriglio et al. (2023).

Cell viability was determined by the MTT assay as described above. Photoprotective efficacy was expressed as the percentage reduction in UV-induced ROS, IL-1α, and NO levels relative to the irradiated untreated controls.

### Histological analysis

Tissue samples of RHE were fixed in 10% neutral-buffered formalin for 48 h, bisected along the central axis, and processed using an automated tissue processor (ASP6025S, Leica Biosystems, Wetzlar, Germany) according to the manufacturer’s instructions. Samples were manually embedded in paraffin with perpendicular orientation to the cut surface to ensure optimal sectioning of the epidermal layers.

From each paraffin block, ten sequential sections of 4 µm thickness were obtained and stained with hematoxylin and eosin (H&E). Slides were scanned using a Leica digital slide scanner (Leica Biosystems, Wetzlar, Germany), and digital images were analyzed with QuPath software (University of Edinburgh, United Kingdom).

Histomorphometric evaluation focused on parameters indicative of UV-induced epithelial damage, including: (i) the number of dyskeratotic keratinocytes (apoptotic or radiation-damaged cells), (ii) the total epithelial thickness, measured separately for the viable cell layers and the keratinized stratum, and (iii) the width of intercellular diastases, corresponding to intercellular clefts at the level of tight junctions.

Enlargement of these diastases, microscopically visible as intercellular “ladders” or “bubbles,” was interpreted as a marker of junctional disruption and epithelial injury.

All measurements were performed on images acquired at 40×, whereas diastases were measured at higher magnification (60×) in five representative regions per sample, avoiding the edges.

### Statistical analysis

All chemical analyses and both cell-free and cell-based assays were performed in three independent experiments, each conducted in triplicate (*n* = 3).

For histological analyses, epithelial thickness values were calculated as the average of three independent measurements, whereas the width of intercellular diastases was determined as the average of five randomly selected areas. Data were expressed as mean ± SD, and statistical significance (*p* < 0.05) was assessed by one-way analysis of variance (ANOVA) followed by the Student–Newman–Keuls and Tukey’s *post hoc* test using SigmaPlot software version 12.0 (Systat Software Inc., San Jose, CA, United States).

## Results

### Raw-material quality control

The starting material, consisting of dried red grape pomace, was subjected to quality control to verify compliance with the purity and safety requirements of the Ph. Eur. for *V. vinifera* L. This step ensured the suitability of the raw material to produce extracts intended for cosmetic applications. Analytical determinations showed a very low residual moisture content (13.55% ± 0.05%), indicating proper stabilization and minimal oxidative degradation risk. ICP-OES analysis confirmed that concentrations of arsenic, cadmium, mercury, and lead were all below 1 mg/kg (Table 1), excluding inorganic contamination.

**TABLE 1:** Quality control parameters of red grape pomace, including chemical and microbiological safety specifications.

Parameter	Specification	Result	Analytical method
Moisture	∼15%	13.55% ± 0.05%	Loss on drying (Ph. Eur.)
Heavy metals			
Lead (Pb)	<3 ppm	0.8 ppm	ICP-OES
Nickel (Ni)	<1 ppm	0.2 ppm	ICP-OES
Cadmium (Cd)	<1 ppm	0.2 ppm	ICP-OES
Mercury (Hg)	<0.1 ppm	<0.05 ppm	ICP-OES
Arsenic (As)	<1 ppm	0.3 ppm	ICP-OES
Microbiology			
TAMC^a^	≤ 10^4^ CFU/g	800 CFU/g	Ph. Eur
TYMC^b^	≤ 10^2^ CFU/g	40 CFU/g	Ph. Eur
*Escherichia coli*	Absent in 1 g	Absent	Ph. Eur
*Salmonella spp.*	Absent in 10 g	Absent	Ph. Eur

^a^ Total aerobic microbial count.

^b^ Total yeast and mold count.

Microbiological analyses revealed total aerobic and fungal counts below 10^2^ and 10^1^ CFU/g, respectively, with *E. coli* and *Salmonella* spp. being absent. Overall, the dried pomace represented a raw material that fully met safety and quality standards, stable and suitable for polyphenol extraction.

### Phytochemical analyses

GPE exhibited a complex and polyphenol-rich phytochemical profile (Table 2).

**TABLE 2:** Preliminary phytochemical screening of the grape pomace extract (GPE). Data represent the mean ± SD of three independent experiments performed in triplicate (*n* = 3).

Test	GPE
Total phenolics (g GAE^a^/100 g DE^b^)	2.64 ± 0.19
Total flavonoids (g RE^c^/100 g DE)	1.67 ± 0.10
Anthocyanins (g MGE^d^/100 g DE)	0.26 ± 0.01
Vanillin index (g CE^e^/100 g DE)	0.86 ± 0.02
Proanthocyanidins (g CcE^f^/100 g DE)	0.280 ± 0.01
Polymerization index^g^	3.07

^a^ GAE, gallic acid equivalents.

^b^ DE, dry extract.

^c^ RE, rutin equivalents.

^d^ MGE, malvidin-3-*O*-glucoside equivalents.

^e^ CE, catechin equivalents.

^f^ CcE, cyanidin chloride equivalents.

^g^ Vanillin index/Proanthocyanidins.

Total phenolics amounted to 2.64 ± 0.19 g GAE/100 g DE, while total flavonoids were 1.67 ± 0.10 g RE/100 g. The vanillin index, indicative of the flavan-3-ol fraction, was 0.86 ± 0.02 g CE/100 g, and total proanthocyanidins were 0.280 ± 0.01 g CcE/100 g.

Monomeric anthocyanins, determined by the pH-differential method, reached 0.26 ± 0.10 g MGE/100 g, confirming that the drying process did not significantly compromise pigment stability. The polymerization index (3.07) indicated a predominance of monomeric flavan-3-ols over oligomeric/polymeric species, suggesting a strong reducing power and major contribution to the overall antioxidant potential of GPE.

According to the phytochemical screening, comprehensive LC-DAD-ESI-MS/MS analysis identified 36 compounds distributed across six structural classes: anthocyanins, lignans, stilbenes, phenylpropanoids, flavonols, and proanthocyanidins (Table 3). Quantitative evaluation of total ion chromatograms revealed a predominance of anthocyanins (52.07%), followed by lignans (20.00%), stilbenes (10.16%), phenylpropanoids (9.83%), flavonols (5.37%), and proanthocyanidins (2.47%).

**TABLE 3:** LC-DAD-ESI-MS/MS analysis of red grape pomace extract (GPE). Retention times, [M + H]^+^/[M–H]^-^ values, MS/MS fragments, and relative TIC peak intensities for identified compounds are reported.

Compound	RT^a^ (min)	MW^b^	[M−H]^+^/[M−H]^−^ (*m/z*)	MS/MS	Peak intensity (*x*10^9^)
Caftaric acid	1.3	312	/311	179–149–135	3.225
Trigallic acid	1.4	474	/473	301–169–125	1.752
Methoxyresveratrol-*O*-sulfate	12.0	322	323/	243–211–107	74.500
Secoisolariciresinol	19.8	362	363/	345–327–193	349.927
*trans*-Resveratrol disulfate	22.6	388	389/	309–229	556.246
Cyanidin-3-*O*-glucoside^c^	24.9	449	449/	287-	696.466
Malvidin-3-*O*-glucoside^c^	26.8	493	493/	331	7.769
Vitisin B	28.7	517	517/	355–340–325	893.093
Vitisin A	30.0	561	561/	399	945.846
Myricetin-*O*-glucuronide	30.1	494	/493	317–299	0.091
Peonidin-*O*-coumaroylglucoside	31.3	609	609/	463–301–147	964.281
Schottenol ferulate	31.5	590	/589	471–193–149	13.192
Malvidin-*O*-glucoside-vinylphenol	31.9	609	609/	463–331–147	935.236
Malvidin-caffeoyl-glucoside	32.7	655	655/	493–331–179	827.334
Quercetin-*O*-rhamnosyl-hexoside	33.8	610	/609	463–301	0.127
Peonidin-*O*-acetylglucoside	34.5	505	505/	463–301–162	746.284
Cyanidin-*O*-coumaroylglucoside	35.4	595	595/	449–287	641.338
Malvidin-caffeoyl-glucoside	35.6	655	/654	493–477–331	0.121
Malvidin-*O*-coumaroylglucoside	36.4	639	693/	493–331–147	466.319
Pelargonidin-*O*-glucosyl-rutinoside	38.3	755	/754	593–431–269	0.296
Malvidin-*O*-glucoside-vinylguaiacol	41.4	639	639/	493–331	448.463
Resveratrol-*O*-sulfate	41.9	308	309/	227–97	443.168
Pyrano-malvidin-coumaroyl-glucoside	42.4	663	663/	517–355–147	446.534
Procyanidin B2-*O*-gallate	43.8	730	731/	712–578–561	463.149
Carboxy-pyrano-malvidin-coumaroylglucoside	44.3	707	707/	561–499–355	419.403
Delphinidin-*O*-coumaroylglucoside	44.6	611	611/	303–465–147	402.362
Myricetin^c^	46.0	318	319/	317–271	397.668
Peonidin-3-*O*-glucoside^c^	50.6	463	463/	301–162	253.087
Myricetin-*O*-arabinoside	52.6	450	451/	319–301	606.973
*trans*-Resveratrol^c^	52.9	228	229/	211–183	520.409
Delphinidin^c^	55.1	303	303/	285–257	498.669
Anhydro-secoisolariciresinol	55.6	344	345/	327–313–151	3393.086
*trans-*Resveratrol-disulfate	58.5	388	389/	309–229–211	308.090
Laricitrin-*O*-hexoside	60.1	494	/493	317	0.494
Malvidin^c^	63.5	331	331/	316–303	596.758
Carboxy-pyrano-cyanidin-*O*-glucoside	72.2	517	517/	355–311–297	1393.967

^a^ RT, retention time.

^b^ MW, molecular weight.

^c^ Verified using commercially available HPLC-grade reference standards (purity ≥98%) purchased from Extrasynthase (Genay, France) or Merck KGaA (Darmstadt, Germany).

Major anthocyanins included malvidin-3-*O*-glucoside, peonidin-3-*O*-glucoside, delphinidin-3-*O*-glucoside, and pyranoanthocyanins (vitisin A/B). Malvidin-3-*O*-glucoside was the most abundant compound (30 mg/100 g dry extract, quantified by HPLC-DAD), and was used for GPE titration.

Stilbene derivatives (*trans*-resveratrol, piceid, and sulfated conjugates) and lignans (secoisolariciresinol, matairesinol, lariciresinol) enriched the extract with additional radical scavenging and anti-inflammatory capacity. Phenylpropanoids (gallic, caffeic, ferulic, *p*-coumaric acids) and flavonols (quercetin, isoquercitrin, rutin, myricetin, kaempferol) reinforced the redox potential and lipid protection. Although less abundant, proanthocyanidins contributed metal-chelating and film-forming stability, completing a balanced and multifunctional plant-complex.

### Antioxidant and anti-inflammatory activities

GPE exhibited concentration-dependent antioxidant effects in complementary assays encompassing single-electron transfer (FRAP), mixed single-electron/hydrogen-atom transfer (TEAC, DPPH), hydrogen-atom transfer and chain-breaking activity in lipid-mimicking systems (ORAC, β-carotene bleaching), as well as Fe^2+^ chelation (ferrozine assay), showing particularly strong results in the FRAP and ORAC assays (Table 4).

**TABLE 4:** Antioxidant and anti-inflammatory properties of the grape pomace extract (GPE). Results are expressed as IC_50_ (µg/mL) with 95% confidence limits. Data are the mean ± SD of three independent experiments performed in triplicate (*n* = 3).

Test	GPE	Standard^a^
	IC_50_ µg/mL (C.L.^b^ 95%)	
Antioxidant activity		
DPPH	336.39 (270.46–418.38)	8.12 (4.28–15.38)^*^
FRAP	87.68 (37.63–204.28)	3.79 (1.69–8.54)^*^
ICA	893.25 (562.06–1419.58)	5.54 (2.44–12.56)^*^
TEAC	199.85 (169.77–248.43)	5.32 (2.86–9.89)^*^
ORAC	8.60 (7.13–10.38)	0.62 (0.14–2.70)^*^
BCB	119.87 (83.79–171.47)	0.44 (0.18–0.82)^*^
Anti-inflammatory activity		
ADA	643.45 (296.67–1395.58)	10.64 (5.01–22.58)^*^
PIA	616.64 (372.49–1021.14)	27.50 (15.17–49.86)^*^

^a^ Trolox for DPPH, FRAP, TEAC, and ORAC; EDTA for ICA; BHT for BCB; and sodium diclofenac for anti-inflammatory assays ADA and PIA.

^b^ Confident limits.

*P<0.001 vs GPE.

Pearson’s linear correlation (*r* ≥ 0.90) indicated a strong positive correlation between total phenolic content and antioxidant activity across all assays, highlighting the synergistic contribution of flavan-3-ols, anthocyanins, and stilbenes.

The anti-inflammatory activity, evaluated by albumin denaturation (ADA) and protease inhibition (PIA), yielded IC_50_ values of 643.45 μg/mL and 616.64 μg/mL, respectively. Although less potent than the reference drug (diclofenac sodium, IC_50_ = 10.64 and 27.50 μg/mL, respectively), GPE demonstrated significant protein-stabilizing and protease-inhibitory effects, supporting its ability to modulate oxidative and inflammatory pathways.

### Photostability

**FIGURE 1:**
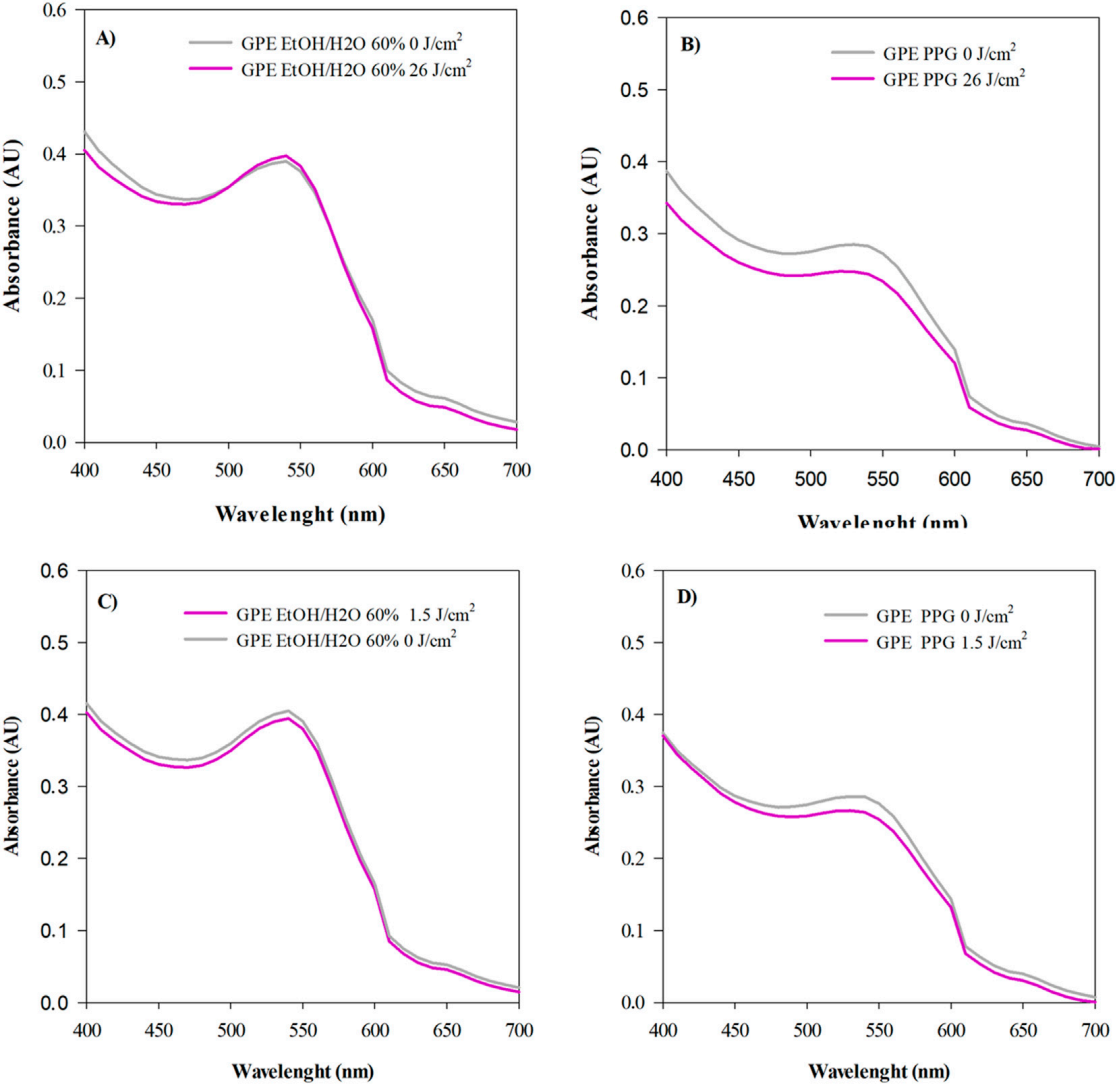
Photostability profiles of the grape pomace extract (GPE, 1 mg/mL) in different solvents after UVA/UVB exposure. UV–Vis spectra of GPE dissolved in ethanol/water (60%, pH 2.0) or propylene glycol following irradiation with UVA (26 J/cm^2^) or UVB (1.5 J/cm^2^). Irradiated spectra are shown in magenta, non-irradiated dark controls in grey. **(A,B)** GPE in ethanol/water and in propylene glycol after UVA exposure; **(C,D)** corresponding samples after UVB exposure. Data are the average of three independent experiments performed in triplicate (*n* = 3).

The photostability of the extract was evaluated by exposing GPE solutions (1 mg/mL) to UVA (26 J/cm^2^) and UVB (1.5 J/cm^2^) radiation, values selected to accurately reproduce the average solar dose to which human skin is exposed under real Mediterranean environmental conditions (Bacqueville and Mavon, 2009) (Figure 1).

The extract exhibited solvent- and wavelength-dependent behaviour. After UVA exposure, spectral degradation was 4.56% in ethanol/water (60%, pH 2.0) and 12.05% in propylene glycol; under UVB exposure, degradation was minimal (3.44% and 3.33%, respectively). These findings indicate a greater susceptibility of GPE to UVA-induced degradation—particularly in propylene glycol—and high intrinsic resistance to UVB radiation.

### Phototoxicity and photoprotection

**FIGURE 2:**
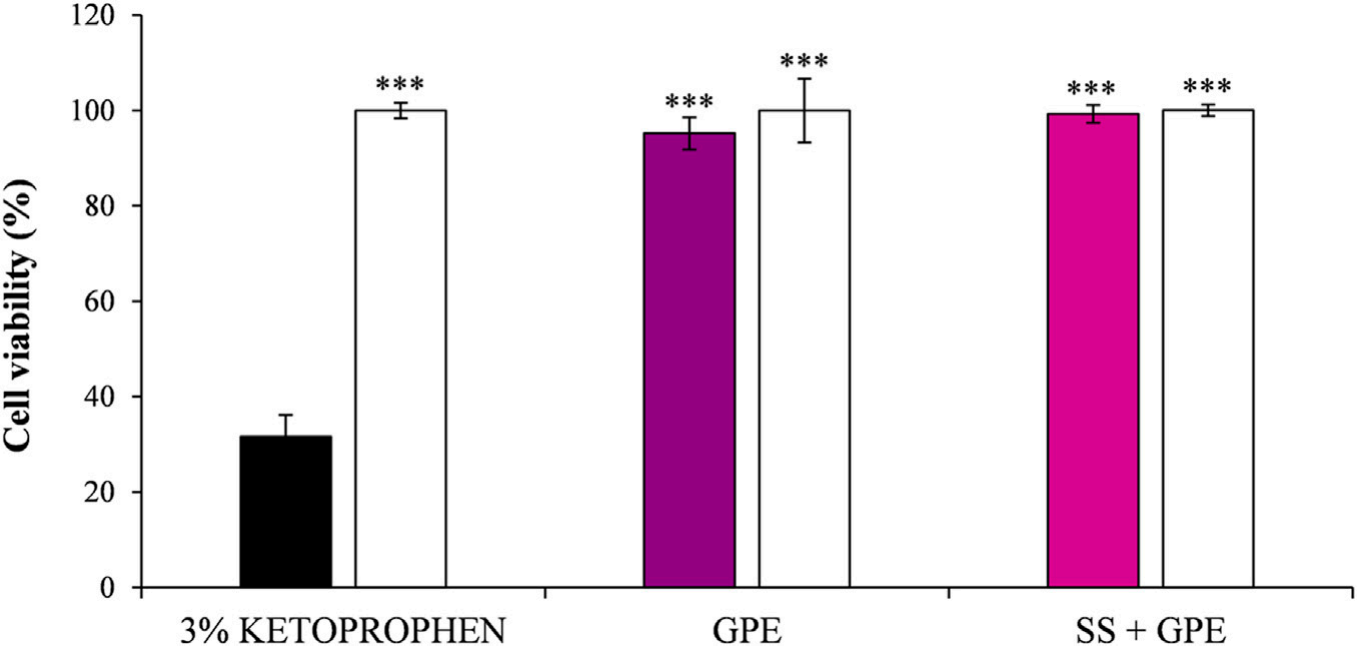
Phototoxicity on reconstructed human epidermis (RHE) under UVA exposure (26 J/cm^2^). Cell viability (%) assessed by the MTT assay in the presence of 3% ketoprofen (positive control, black bar), grape pomace extract (GPE), and SPF 50+ sunscreen supplemented with GPE (SS + GPE). White bars: non-irradiated controls; deep magenta bar (GPE) and light magenta bar (SS + GPE): irradiated samples. Data are expressed as mean ± SD of three independent experiments performed in triplicate (n = 3). One-way ANOVA followed by the Student–Newman–Keuls *post hoc* test: ^***^ *p* < 0.001 vs. positive control (3% ketoprofen).

The phototoxicity potential of GPE was assessed under UVA irradiation (26 J/cm^2^), while its photoprotective effect was evaluated on RHE exposed to UVA (26 J/cm^2^) and UVB (1.5 J/cm^2^) irradiation. Neither GPE (5 mg/mL in propylene glycol) nor the SPF 50+ sunscreen formulation containing 0.5% GPE (SS + GPE) significantly affected cell viability (>90%) compared to non-irradiated controls, whereas 3% ketoprofen markedly reduced the cell viability (∼70%, *p* < 0.001) (Figure 2).

**FIGURE 3:**
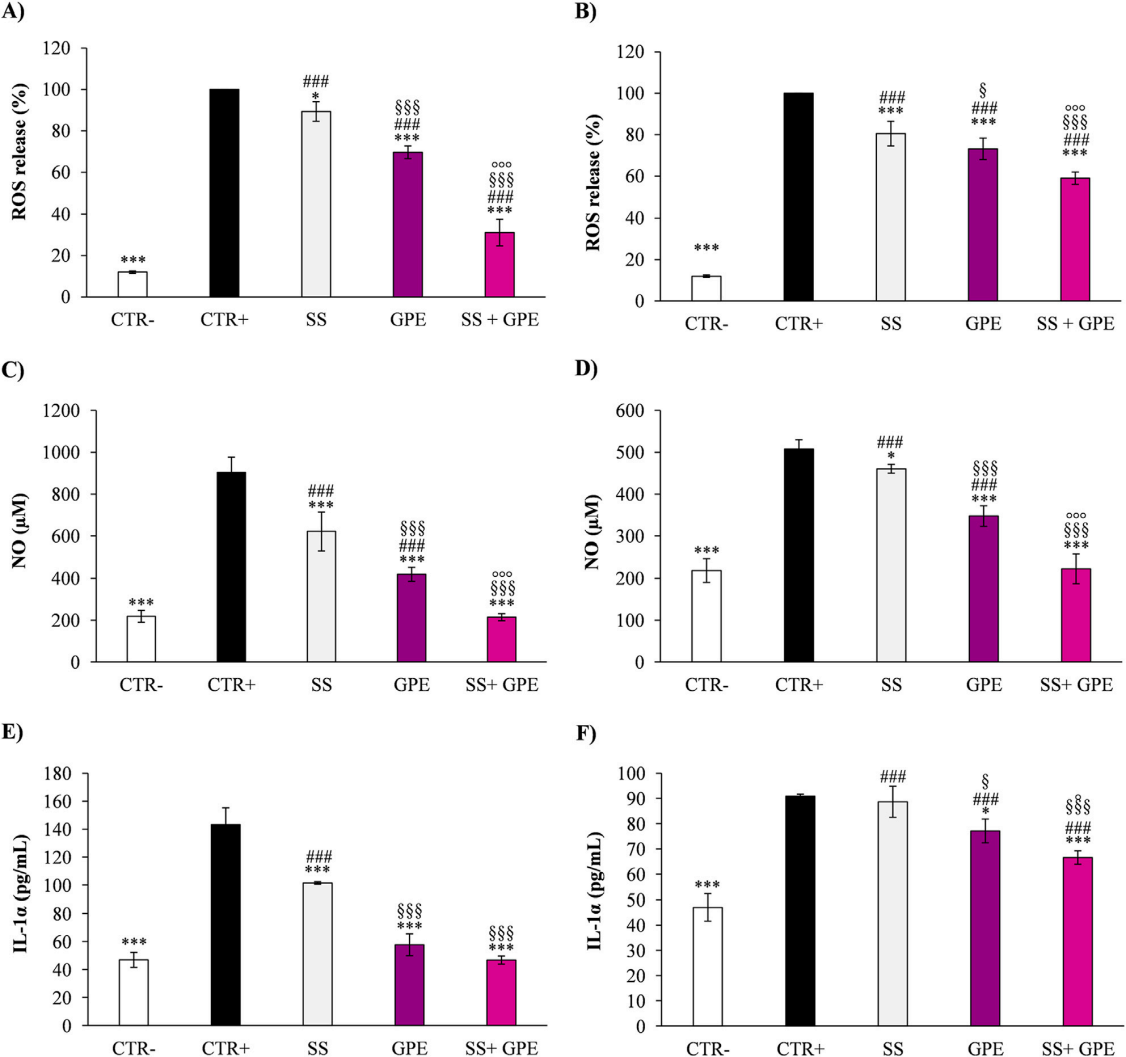
Photoprotective effects of grape pomace extract (GPE, deep magenta bars) and base sunscreen (SS, grey bars), alone or combined (SS + GPE, light magenta bars), on oxidative and inflammatory markers in RHE. Effects on intracellular reactive oxygen species (ROS), nitric oxide (NO), and interleukin-1α (IL-1α) after exposure to UVA (panels **(A,C,E)**) or UVB (panels **(B,D,F)**). CTR–: untreated, non-irradiated control; CTR+: untreated, irradiated control. Data are mean ± SD of three independent experiments performed in triplicate (*n* = 3). One-way ANOVA followed by the Student–Newman–Keuls *post hoc* test: ^*^ *p* < 0.05, ^***^ *p* < 0.001 vs. CTR+; ^###^ *p* < 0.001 vs. CTR–; ^§^ *p* < 0.05, ^§§§^ *p* < 0.001 vs. LS; °*p* < 0.05,   °°°*p* < 0.001 vs. GPE.

GPE also markedly reduced oxidative and inflammatory biomarkers: ROS (−45%, *p* < 0.001), NO (−39%, *p* < 0.001), and IL-1α (−42%, *p* < 0.001) relative to irradiated controls. Under UVA exposure, the SS + GPE formulation produced significantly stronger effects (*p* < 0.001) than GPE alone for all markers except IL-1α. Under UVB exposure, SS + GPE also showed significantly stronger effects than GPE, with *p* < 0.001 for ROS and NO and *p* < 0.05 for IL-1α (Figure 3).

The base sunscreen (SS) exhibited a modest intrinsic antioxidant and soothing activity (∼15–20%), plausibly attributable to the presence of lipid emollients, tocopherol, and UV filters with partial free-radical scavenging capacity. However, its combination with GPE produced a clear enhanced effect, maintaining cell viability at levels comparable to the non-irradiated control (*p* < 0.001 vs. irradiated group).

**FIGURE 4:**
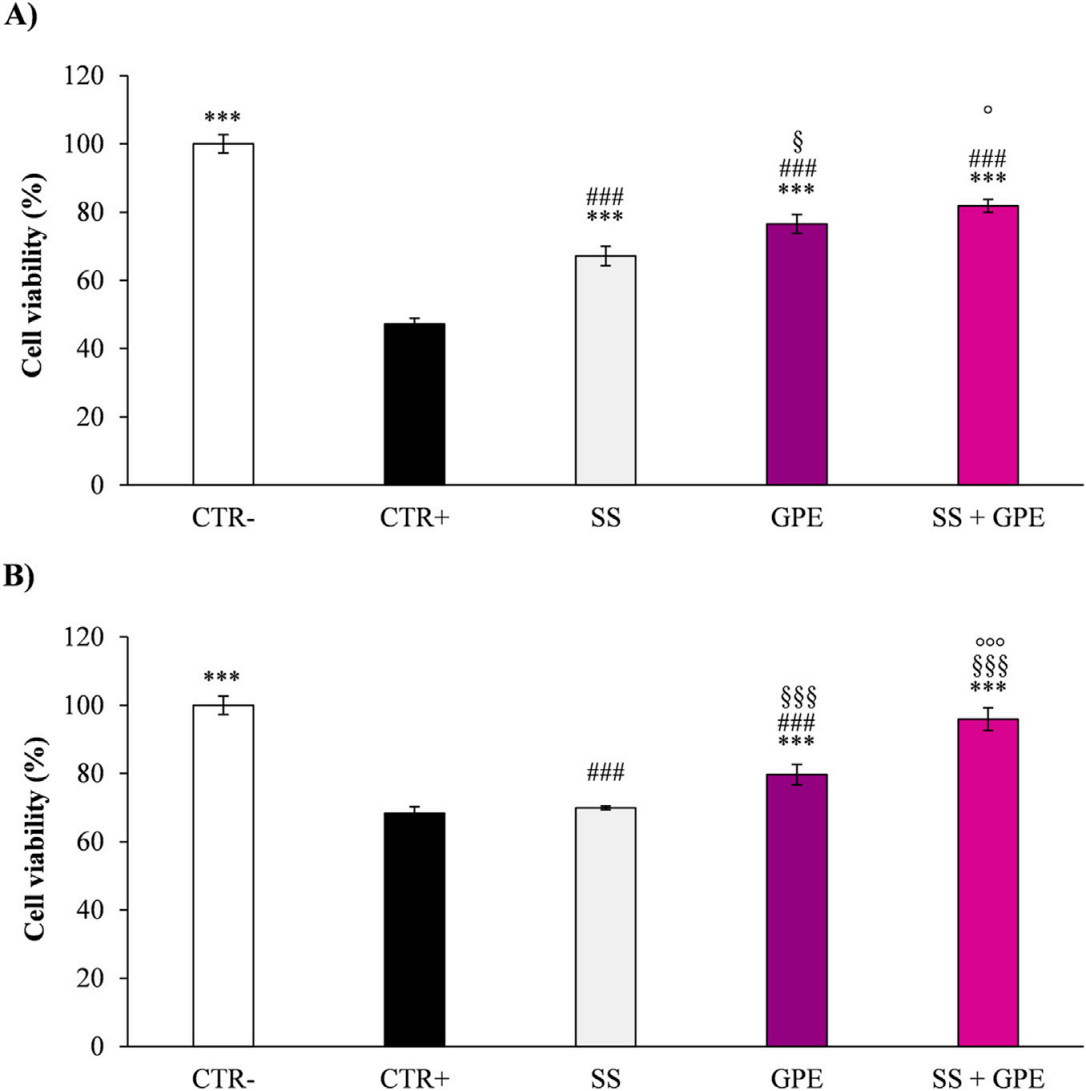
Cell viability (MTT) in RHE exposed to UVA (26 J/cm^2^; panel **(A)** or UVB (1.5 J/cm^2^; panel **(B)** and treated with base sunscreen (SS, grey bar), grape pomace extract (GPE, deep magenta bar), alone or combined (SS + GPE, light magenta bar). CTR–: untreated, non-irradiated control (white bar); CTR+: untreated, irradiated control (black bar). Data are mean ± SD of three independent experiments performed in triplicate (*n* = 3). One-way ANOVA followed by the Student–Newman–Keuls *post hoc* test: ^*^ *p* < 0.05, ^***^ *p* < 0.001 vs. CTR+; ^###^ *p* < 0.001 vs. CTR–; ^§^ *p* < 0.05, ^§§§^ *p* < 0.001 vs. LS;°*p* < 0.05,   °°°*p* < 0.001 vs. GPE.

Mechanistically, UVA (320–400 nm) penetrates deeper and induces oxidative stress mainly through ROS generation. In this context, GPE—alone or formulated—significantly decreased intracellular ROS and NO (>50%, *p* < 0.001) and maintained cell viability (Figure 4), consistent with the UVA-absorbing and radical-scavenging properties of anthocyanins, flavonols, and stilbenes (Hübner et al., 2019). UVB (280–320 nm), being more energetic, caused direct DNA and membrane damage with cytokine release (IL-1α) (Vechtomova et al., 2021); GPE remained protective, with the formulated version outperforming the pure extract, likely due to enhanced photostability and surface retention provided by the cosmetic matrix.

In summary, GPE acts as a multifunctional photoprotective agent, displaying greater efficacy against UVA-driven oxidative stress and significant protection from UVB-induced inflammatory damage. The synergy between antioxidant and anti-inflammatory pathways, enhanced by the cosmetic formulation, supports the potential of GPE as a safe, photostable, and functional natural ingredient for broad-spectrum skin photoprotection.

### Histopathological findings

Digital images of RHE samples were analyzed using QuPath software to perform standardized morphometric evaluations, including epithelial thickness, stratum corneum thickness, number of dyskeratotic (sunburn) cells, and width of intercellular diastases.

**FIGURE 5:**
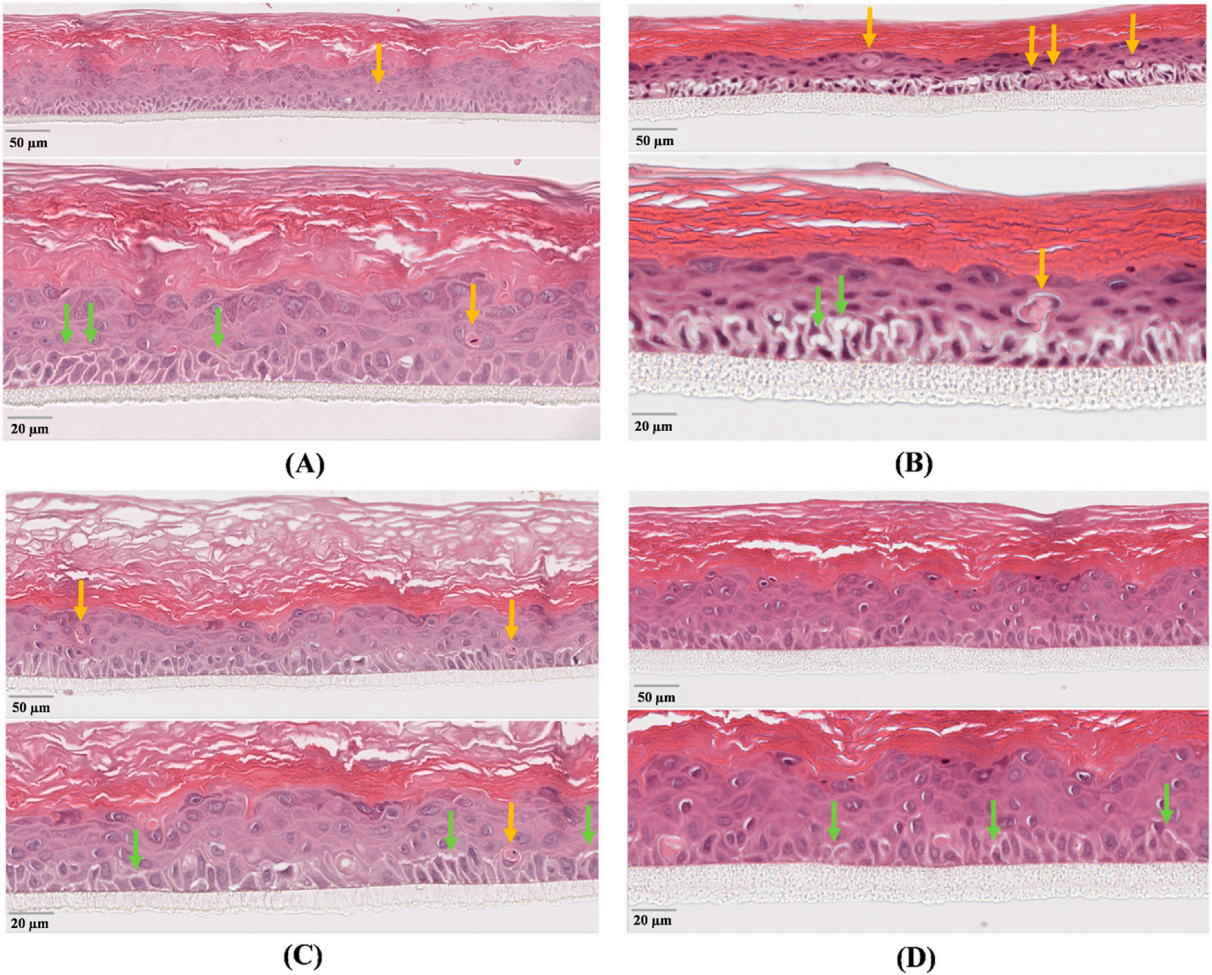
Histological analysis of reconstructed human epidermis (HRE) following UVA exposure. Figure shows micrographs of: **(A)** untreated non-irradiated control (CTR–); **(B)** untreated irradiated control (CTR+); **(C)** tissue treated with the grape pomace extract (GPE); **(D)** tissue treated with formulation (SS + GPE). Representative H&E-stained sections are shown at two magnifications (low and high) for each treatment, with scale bars corresponding to 50 µm and 20 μm, respectively. Yellow arrows indicate dyskeratotic cells, whereas green arrows highlight intercellular diastasis; the width of intercellular separation was used as a morphometric indicator of epithelial damage. All samples were observed under identical magnification (40×, upper micrograph of each panel), whereas diastases were measured at higher magnification (60×, lower micrograph of each panel) in five representative regions per sample.

**FIGURE 6:**
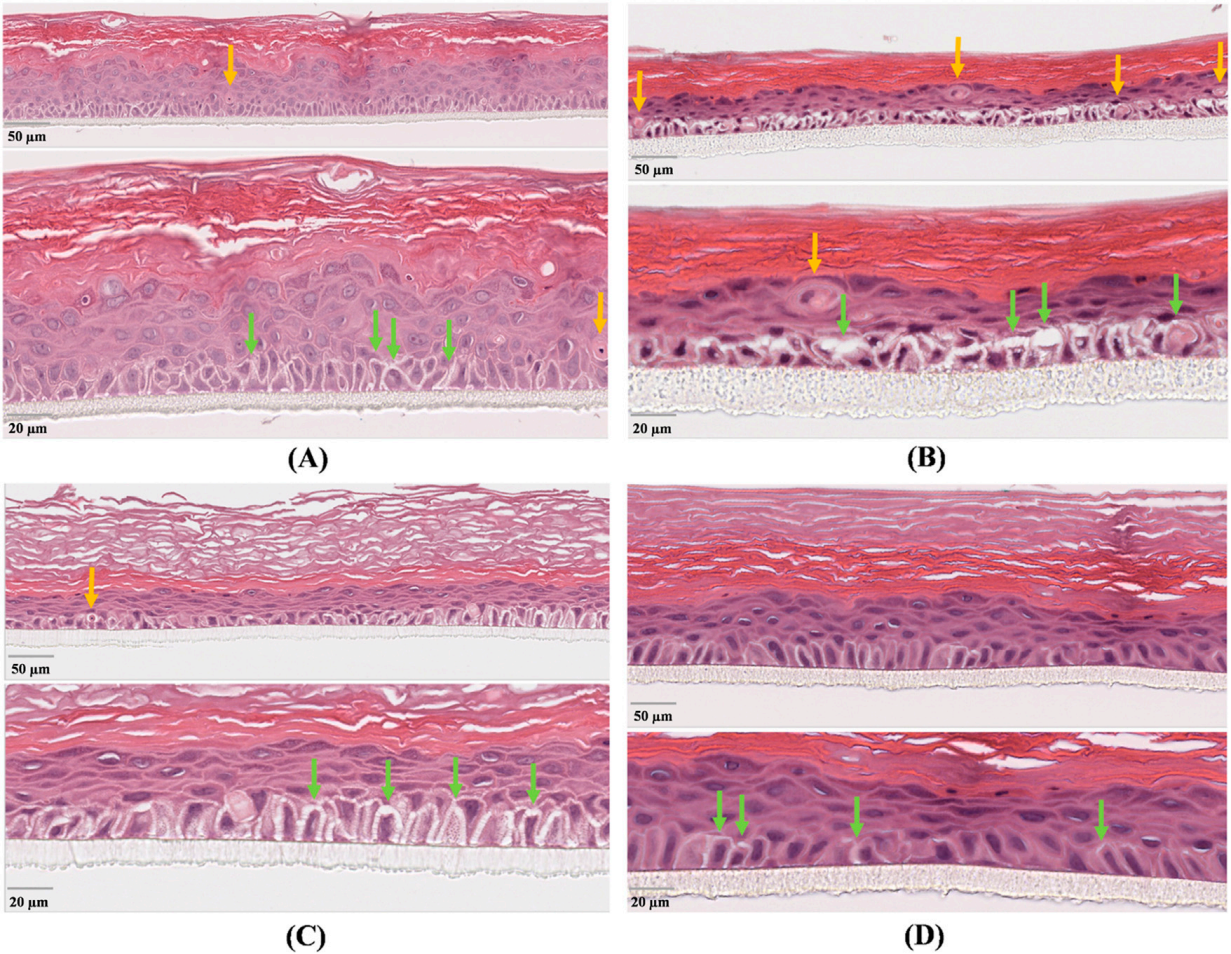
Histological analysis of reconstructed human epidermis (HRE) following UVB exposure. Figure shows micrographs of: **(A)** untreated non-irradiated control (CTR–); **(B)** untreated irradiated control (CTR+); **(C)** tissue treated with the grape pomace extract (GPE); **(D)** tissue treated with formulation (SS + GPE). Representative H&E-stained sections are shown at two magnifications (low and high) for each treatment, with scale bars corresponding to 50 µm and 20 μm, respectively. Yellow arrows indicate dyskeratotic cells, whereas green arrows highlight intercellular diastasis; the width of intercellular separation was used as a morphometric indicator of epithelial damage. All samples were observed under identical magnification (40×, upper micrograph of each panel), whereas diastases were measured at higher magnification (60×, lower micrograph of each panel) in five representative regions per sample.

The negative non-irradiated controls (Figures 5A, 6A) displayed normal histoarchitecture, with thicker epithelium (72 and 74 µm) and keratin layer (91 and 93 µm), very few dyskeratotic cells (1 in both cases), and narrow diastases (2.2 and 2.4 µm). Conversely, the positive controls irradiated both with UVA (Figure 5B) and UVB (Figure 6B) exhibited marked epithelial thinning (47 and 49 µm) and reduced keratin thickness (46 and 44 µm), associated with numerous dyskeratotic cells (4/700 µm in both cases) and wide intercellular spaces (5.8 and 6.1 µm), consistent with severe epithelial damage and loss of cohesion.

After UVA exposure, GPE treatment (Figure 5C) partially restored epithelial morphology, with a thicker viable layer (70 µm vs. 47 μm in control UVA) and a substantially increased keratin layer (110 µm). The number of dyskeratotic cells was halved (2 vs. 4), and mean diastasis width decreased from 5.8 µm to 3.1 µm, indicating attenuation of UVA-induced damage and partial recovery of intercellular cohesion.

The cosmetic formulation (SS + GPE, Figure 5D) exhibited an almost normal histological pattern, with epithelial and keratin thickness close to control values (71 µm and 75 μm, respectively), complete absence of dyskeratotic cells, and minimal diastases (1.5 µm). These findings confirm an enhanced protective effect, with full recovery of epithelial organization and normalization of intercellular junctions.

Under UVB exposure, GPE alone (Figure 6C) showed a partially preserved morphology, with thinner epithelium (41 µm) but thicker keratin (82 µm), few dyskeratotic cells (1), and moderately enlarged diastases (3.2 µm), suggesting reduced apoptosis but persistent structural stress due to the higher energy of UVB radiation. In contrast, the combined treatment (SS + GPE, Figure 6D) restored near-normal morphology, with epithelial and keratin thickness of 47 µm and 65 μm, absence of dyskeratotic cells, and diastases comparable to the non-irradiated control (1.5 µm).

A quantitative summary of morphometric parameters is reported in Table 5, showing consistent trends across all experimental conditions. Both UVA and UVB radiations induced pronounced epithelial damage, yet protective treatments—particularly SS + GPE—significantly improved all histological markers, restoring epithelial and keratin thickness, intercellular cohesion, and cellular integrity to physiological levels. Intercellular diastases, a sensitive marker of barrier disruption, were drastically reduced by the combined treatment, while dyskeratotic cells were almost completely absent. The keratin-to-viable-epithelium ratio normalized, reflecting balanced differentiation and tissue regeneration.

**TABLE 5:** Interpretative summary of histological parameters in reconstructed human epidermis after UVA and UVB exposure. The table summarizes the qualitative evaluation of intercellular cohesion, apoptosis (dyskeratotic cells), keratinization response, and overall histological outcome in reconstructed human epidermis (HRE) following exposure to UVA and UVB radiation. Descriptive assessment was based on the histomorphological features observed in H&E-stained sections and expressed using semi-quantitative terms. The overall epithelial outcome was interpreted in relation to the degree of intercellular cohesion and the number of dyskeratotic cells, providing a comparative view of tissue integrity and photoprotective effects across treatments.

Treatment	Epithelial damage	Intercellular cohesion	Apoptosis	Keratinization response	Overall effect
CTR–^a^	Absent	Intact	Minimal	Normal	Physiological
UVA-irradiated CTR+^b^	Marked	Compromised	Marked	Reduced	Acute damage
UVA-irradiated GPE^c^	Moderate	Partially restored	Reduced	Adaptive hyperkeratosis	Partial protection
UVA-irradiated SS + GPE^d^	Minimal	Normalized	Absent	Physiological	Marked protection
UVB-irradiated CTR+^e^	Marked	Compromised	Marked	Reduced	Acute damage
UVB-irradiated GPE^f^	Moderate	Partially restored	Low	Hyperkeratosis	Partial protection
UVB-irradiated SS + GPE^g^	Minimal	Normalized	Absent	Physiological	Marked protection

^a^ Untreated, non-irradiated tissue (CTR–).

^b^ Untreated UVA-irradiated tissue (CTR+).

^c^ UVA-irradiated tissue treated with grape pomace extract (GPE).

^d^ UVA-irradiated tissue treated with sunscreen + grape pomace extract (SS + GPE).

^e^ Untreated UVB-irradiated tissue (CTR+).

^f^ UVB-irradiated tissue treated with grape pomace extract (GPE).

^g^ UVB-irradiated tissue treated with sunscreen + grape pomace extract (SS + GPE).

Overall, these findings demonstrate a structural and functional photoprotective effect of GPE, which was further potentiated when incorporated into the O/W emulsion of SS. The presence of lipid emollients, vegetable oils, tocopherol, and organic UV filters in the formulation likely enhanced the formation of a uniform protective film over the epidermal surface, improving adhesion, controlling the diffusion and penetration of active compounds, and stabilizing the polyphenolic fraction. This enhanced interaction preserved intercellular cohesion, promoted keratinocyte differentiation, and maintained overall epidermal integrity under UVA/UVB-induced stress.

## Discussions

The present study highlights the remarkable potential of the grape pomace extract (GPE) as a complex and multifunctional source of bioactive polyphenols. The LC-DAD-ESI-MS/MS characterization enabled the identification of thirty-six secondary metabolites distributed across six main structural classes—anthocyanins, lignans, stilbenes, phenylpropanoids, flavonols, and proanthocyanidins—defining a rich and well-balanced phytochemical profile. The anthocyanin fraction, which predominated, was mainly composed of malvidin-3-*O*-glucoside, used as a phytochemical marker for extract titration. These findings are fully consistent with the evidence reported by Delić et al. (2024), who described malvidin-3-*O*-glucoside as the major anthocyanin pigment in *Vitis vinifera* L. pomace, together with peonidin, delphinidin, and petunidin, confirming its crucial role in photo-absorbing and antioxidant properties. The same authors also pointed out that enzymatic and chemical transformations occurring during vinification can enrich the anthocyanin profile of pomace compared to must, making it a high-value functional matrix.

The consistent presence of lignans (20%) observed in the present study further broadens the chemical spectrum compared to previously reported pomace profiles. According to Karastergiou et al. (2024), lignans represent an emerging class of compounds in wine-making by-products, characterized by high oxidative stability and potential protective effects at the cellular level. Although the coexistence of multiple phenolic subclasses may contribute to the overall functional complexity of the extract, the present study did not directly investigate interaction or stabilization mechanisms among isolated compound classes. Therefore, no conclusion regarding synergistic photochemical stabilization can be drawn from the current dataset.

This point is especially relevant in dermocosmetic contexts, where stabilization of the epidermal lipid matrix and prevention of UV-induced lipid peroxidation represent essential protective mechanisms.

Similarly, the stilbene fraction—dominated by trans-resveratrol and its sulfate derivatives—confirms the ability of grape pomace to retain molecules typical of the fresh fruit, in agreement with Chedea et al. (2022) and Guaita et al. (2023). The latter demonstrated that, despite fermentation processes, pomace preserves a surprisingly rich qualitative composition in resveratrol, quercetin, and glycosylated anthocyanins, all endowed with notable antioxidant and stabilizing capacities.

As also reported by Karastergiou et al. (2025), the phenolic composition of grape pomace varies significantly depending on cultivar and pedoclimatic conditions but consistently maintains a high phytochemical diversity and robust antioxidant potential, especially in red French cultivars, confirming the universal value of this matrix as a sustainable source of bioactive polyphenols.

From a biological standpoint, GPE showed pronounced antioxidant activity, confirmed by consistent results across different spectrophotometric and spectrofluorimetric assays. The IC_50_ values obtained were comparable to those reported for polyphenol-rich pomace extracts in recent studies by Ferri et al. (2023) and Bocsan et al. (2022), confirming high radical-scavenging efficiency and strong reducing power. The positive correlation between total phenolic content and antioxidant capacity observed in this study reflects the complementary contribution among the various phenolic classes, as previously evidenced by Chedea et al. (2022) and Radulescu et al. (2024). According to these authors, anthocyanins and stilbenes cooperate to maintain intracellular redox homeostasis, with flavonols and phenylpropanoids playing a modulatory role by stabilizing semiquinone radicals generated during redox processes.

Such cooperative activity is consistent with the multifunctional outcomes observed in subsequent anti-inflammatory and epidermal protection assays.

The anti-inflammatory activity observed in this study showed significant, concentration-dependent effects in both the albumin denaturation and protease inhibition assays. Although the extract showed consistent multi-target activity, the study design does not allow attribution of synergistic interactions among individual phenolic subclasses, as only the whole extract was tested.

Comparable results were reported by Draghici-Popa et al. (2024), who demonstrated dose-dependent anti-inflammatory activity of grape pomace extracts in cell models of induced inflammation, with reductions in IL-6 and TNF-α and increased expression of endogenous antioxidant enzymes. Moreover, Radulescu et al. (2024) observed that phenolic extracts of grape pomace, in addition to antioxidant and anti-inflammatory effects, display selective antimicrobial activity against pathogenic skin microorganisms, suggesting additional dermoprotective and preservative potential useful for cosmetic applications.

These converging effects underline that GPE maintains epidermal functional balance by modulating both oxidative and inflammatory stress pathways, which are tightly interconnected under UV exposure conditions.

Consistent findings were also reported by Grosu et al. (2024), who showed that hydroalcoholic extracts from autochthonous Romanian grape pomace varieties exhibit polyphenol-rich profiles with high anthocyanin and flavonol content, associated with significant antioxidant and antimicrobial properties, further supporting the use of grape pomace as a natural multifunctional ingredient for protective and preservative cosmetic formulations.

From a technological perspective, the present study confirms that propylene glycol represents an excellent cosmetic vehicle for stabilizing polyphenolic phytocomplexes. Photostability tests demonstrated that the extract retained more than 90% of its initial absorbance after exposure to UVA (26 J/cm^2^) and UVB (1.5 J/cm^2^) irradiation, parameters selected to reproduce realistic solar exposure conditions (Hübner et al., 2020; IARC Working Group on the Evaluation of Carcinogenic Risks to Humans, 2012). To ensure physiologically relevant irradiation conditions, the UVA and UVB doses were obtained by using the total UV exposure reported by Hübner et al. (2020) as a reference and partitioning it according to the natural distribution of solar ultraviolet radiation at the Earth’s surface, where approximately 95% corresponds to UVA and about 5% to UVB (IARC Working Group on the Evaluation of Carcinogenic Risks to Humans, 2012). This approach allowed the application of environmentally consistent and biologically meaningful UVA and UVB doses in experimental treatments.

This behaviour may be attributed to the high compatibility of propylene glycol with polar molecules, which minimizes oxidation and photo-isomerization processes of anthocyanins. Furthermore, the formulation containing GPE exhibited a superior photoprotective profile compared to the base formulation, in line with previous reports indicating that lipid-based vehicles can improve the cutaneous deposition and persistence of polyphenols (Valenzuela-Bustamante et al., 2025). The cited study by Valenzuela-Bustamante et al. (2025) demonstrated that incorporating grape pomace extracts into lipid emulsions improved both thermal and photochemical stability, reduced anthocyanin degradation, and enhanced UV-protective efficacy. Similarly, Lopes et al. (2025) demonstrated that cosmetic formulations containing grape pomace polyphenols increase skin antioxidant capacity and protect epidermal cells from photo-induced stress through a prolonged barrier effect mediated by the formation of polyphenolic microfilms on the stratum corneum.

In agreement with the biochemical and cellular findings, histological analysis provided morphological confirmation of the photoprotective efficacy of GPE. The preservation of epithelial integrity, the reduction in dyskeratotic cells, and the normalization of keratinization collectively demonstrate that GPE confers effective structural protection against UV-induced injury. The improved intercellular cohesion and reduced diastasis observed in treated samples are consistent with antioxidant and anti-inflammatory protection mechanisms previously documented for grape-derived polyphenols (Salucci et al., 2017; Guo et al., 2024). Notably, the superior morphological preservation observed with the combined formulation (SS + GPE) compared to GPE alone highlights the importance of the lipidic vehicle, which likely promotes better diffusion, bioavailability, and retention of active compounds at the epidermal surface. Taken together, these findings reinforce the concept that GPE acts as a multifunctional extract capable of preserving both the structural and functional homeostasis of the epidermis under UVA/UVB exposure, thus supporting its potential use in advanced dermocosmetic formulations for broad-spectrum photoprotection (Verma et al., 2024).

Beyond biological activity, one of the major strengths of this work lies in the rigorous quality control of the raw matrix. The dried grape pomace underwent comprehensive chemical, microbiological, and safety assessments—including determination of heavy metals, microbial load, and residual moisture—ensuring suitability for cosmetic and nutraceutical applications. This approach, rarely addressed in academic studies, represents an essential prerequisite for industrial transferability. In this regard, our findings align with the recommendations of Wang et al. (2024) and Castillo et al. (2022), who emphasized that the valorization of agri-food by-products must necessarily include safety evaluation and optimization of extraction processes following “green” and sustainable principles. Implementing such quality-control protocols not only improves reproducibility but also ensures compliance with European cosmetic and nutraceutical regulations.

The phytochemical complexity and high stability observed in GPE are consistent with recent studies by Almanza-Oliveros et al. (2024), who analyzed the enhanced behaviour of anthocyanins, stilbenes, and flavonols in complex matrices, demonstrating that the overall bioactivity of such systems cannot be ascribed to the sum of their individual components but to the maintenance of a multifactorial redox balance. This concept, also expressed by Mora-Garrido et al. (2025), highlights that the functionality of complex phytochemical matrices derives from the co-occurrence of multiple compound classes rather than isolated constituents; however, the present study did not experimentally dissect the contribution of individual phenolic subclasses.

In this context, the present study fits perfectly within the paradigm of circular economy and sustainable bioeconomy, demonstrating that a by-product traditionally considered waste can be recovered, characterized, and reused in high-value applications. According to Lopes et al. (2025) and Mora-Garrido et al. (2025), the valorization of grape pomace as a source of bioactive compounds represents one of the most promising strategies to reduce the environmental impact of the wine industry while creating new economic opportunities for the cosmetic and nutraceutical sectors. The concept of enological “up-cycling,” widely discussed by Karastergiou et al. (2024) and Valenzuela-Bustamante et al. (2025), finds here a concrete application through an integrated experimental approach combining raw-material quality control, advanced chemical characterization, and multiparametric biological validation.

Indeed, in the context of existing literature, the distinctive contribution of this work lies in the evaluation of a chemically standardized grape pomace extract in a RHE model exposed to controlled and physiologically relevant doses of both UVA and UVB radiation. While previous studies have described the antioxidant or anti-inflammatory potential of grape pomace using cell-free systems or monolayer cell cultures, no data were available on its ability to preserve epidermal morphology and function within a three-dimensional human skin equivalent. The present findings show that GPE effectively mitigates structural hallmarks of UV-induced damage—including epithelial disorganization, dyskeratosis, and intercellular separation—while preserving keratinization and tissue cohesion and modulating oxidative and inflammatory stress pathways. This integrated evidence highlights, for the first time, the broad-spectrum photoprotective behaviour of a grape pomace–derived phytocomplex in a physiologically relevant epidermal model, reinforcing its potential for dermocosmetic applications.

Beyond its biological activity, a further distinctive aspect of this work lies in the rigorous quality control applied to the starting material, carried out according to strict safety and traceability criteria rarely implemented in academic research on agro-industrial by-products. This approach validated the dried grape pomace not only as an experimental model but also as a genuinely transferable ingredient for cosmetic and nutraceutical use, fully consistent with the principles of circular economy and environmental sustainability. This represents a key translational advantage compared to previous studies focused solely on laboratory-scale extraction or chemical profiling.

Although the study presents certain limitations—such as the need to elucidate molecular mechanisms and to confirm the findings in clinical or cosmetic settings—it provides a solid foundation for future developments. In this perspective, the implementation of sustainable extraction technologies, phytochemical standardization, and clinical validation of safety and efficacy represent essential steps toward the industrial consolidation of grape pomace as a biofunctional resource.

Overall, GPE emerges as a sustainable and multifunctional bioactive ingredient with demonstrated antioxidant, anti-inflammatory, and photoprotective properties, paving the way for its integration into next-generation dermocosmetic formulations designed for broad-spectrum epidermal protection.

## Conclusions

This study demonstrated that red grape pomace, traditionally considered a low-value by-product, is a rich and multifunctional source of phenolic compounds with clear biological relevance. Its well-balanced phytochemical profile—dominated by anthocyanins and complemented by lignans, stilbenes, and flavonols—was reflected in strong antioxidant and anti-inflammatory effects. Importantly, the extract provided broad-spectrum photoprotection in a reconstructed human epidermis model exposed to physiologically relevant UVA and UVB doses, supporting its potential use as a sustainable ingredient for dermocosmetic applications.

## Data Availability

The original contributions presented in the study are included in the article/supplementary material, further inquiries can be directed to the corresponding author.
